# Distribution of human-pathogenic *Cryptosporidium* spp., *Giardia duodenalis*, and *Enterocytozoon bieneusi* in crab-eating macaques in China

**DOI:** 10.3389/fmicb.2025.1641632

**Published:** 2025-07-21

**Authors:** Huilin Zhang, Huiyang Chen, Chaoyue He, Wenchao Li, Falei Li

**Affiliations:** ^1^Anhui Province Key Laboratory of Embryo Development and Reproductive Regulation, College of Biological and Food Engineering, Fuyang Normal University, Fuyang, China; ^2^Anhui Province Key Laboratory of Animal Nutritional Regulation and Health, College of Animal Science, Anhui Science and Technology University, Fengyang, China

**Keywords:** *Cryptosporidium* spp., *Giardia duodenalis*, *Enterocytozoon bieneusi*, crab-eating macaque, zoonosis, China

## Abstract

**Introduction:**

The positive rates and genetic identity of *Cryptosporidium* spp., *Giardia duodenalis* (*G. duodenalis*), and *Enterocytozoon bieneusi* (*E. bieneusi*) were unclear in crab-eating macaques in Suzhou and Beijing, China.

**Methods:**

A total of 504 fecal samples were collected from crab-eating macaques on commercial farms in Beijing and Suzhou, China. The extracted DNA was analyzed for *Cryptosporidium* spp. and *E. bieneusi* by nested PCR and sequence analysis of the small subunit rRNA (*SSU* rRNA) gene and the internal transcribed spacer (ITS) gene, respectively. The *G. duodenalis* was detected by nested PCR targeting *β*-giardin (*bg*) gene, glutamate dehydrogenase (*gdh*) gene, and triosephosphate isomerase (*tpi*) gene. The *C. hominis* identified were further subtyped by nested PCR and sequence analysis of the 60 kDa glycoprotein (*gp60*) gene.

**Results:**

All 504 fecal samples collected from crab-eating macaques, the detection rates of *Cryptosporidium* spp., *G. duodenalis*, and *E. bieneusi* were 11.9% (60/504), 5.6% (28/504), and 4.6% (23/504), respectively. The 15.1% (44/292) detection rate of *Cryptosporidium* spp. from crab-eating macaques in Suzhou was significantly higher than that in Beijing (2.8%; 6/212; *χ*^2^ = 20.6, *df* = 1, *p* < 0.0001). The detection rates of *Cryptosporidium* spp. and *G. duodenalis* were significant different between <2 months old animals and >24 months old animals (*χ*^2^ = 104.7, *df* = 1, *p* < 0.0001; *χ*^2^ = 6.6, *df* = 1, *p* = 0.0104). In contrast, there was no significant different in the detection rate of *E. bieneusi* in two age groups (*χ*^2^ = 2.2, *df* = 1, *p* = 0.1360). A total of one *Cryptosporidium* species, one *G. duodenalis* assemblage B, and 4 *E. bieneusi* genotypes have been identified, including *C. hominis* (*n* = 60), assemblage B (*n* = 28), CM1 (*n* = 14), Peru8 (*n* = 5), D (*n* = 3), and Type IV (*n* = 1). Among 60 *C. hominis* samples, five subtypes of five subtype families were successfully identified at the *gp60* gene: IbA13G4 (*n* = 27), InA26 (*n* = 3), IfA17G2R3 (*n* = 3), IiA17 (*n* = 3), and IeA11G3T3 (*n* = 2).

**Discussion:**

The results indicate that known zoonotic *Cryptosporidium* spp., *G. duodenalis*, and *E. bieneusi* are prevalent in crab-eating macaques. The crab-eating macaques could play a potential role in the zoonotic transmission of pathogens to humans.

## 1 Introduction

*Cryptosporidium* spp., *Giardia duodenalis* (*G. duodenalis*), and *Enterocytozoon bieneusi* (*E. bieneusi*) are common zoonotic protozoan pathogens in humans, non-human primates, and ruminants, causing moderate-to-severe diarrhea in animals (Li W. et al., 2019; Cai et al., 2021; Guo et al., 2021b). These three gastrointestinal pathogens are mainly transmitted through food-borne transmission and water-borne transmission (Xiao, 2010). In non-human primates, crab-eating macaques are very similar to humans in physiology and genetics and are used in human disease research and drug experiments (Zhang et al., 2014). In the laboratory, crab-eating macaques have close contact with humans. Therefore, these animals could become potential hosts for zoonotic *Cryptosporidium* spp., *G. duodenalis*, and *E. bieneusi*.

So far 47 *Cryptosporidium* species and more than 120 *Cryptosporidium* genotypes have been recognized in humans and animals, and most of them are host-adapted (Ryan et al., 2021). Among them, *C. hominis* has a narrower host range and mainly detect in humans and non-human primates (Huang et al., 2025). Although, *C. parvum* has a broad host range and is rarely found in non-human primates (Feng et al., 2018). More than 10 subtype families of *C. hominis* have been recognized based on sequence analysis of the 60 kDa glycoprotein (*gp60*) gene (Xiao and Feng, 2017). Among these subtype families, Ia, Ib, Ie, and Ii were only found in humans and non-human primates (Feng and Xiao, 2017). By contrast, In subtype family was only found in crab-eating macaques in Hainan (Chen et al., 2019). These subtype families of *C. hominis* differ in virulence, with subtype IbA10G2 widely distributed in both industrialized and developing countries (Bouzid et al., 2013). Subtype IbA10G2 always causes *C. hominis*-associated outbreaks in industrialized countries (Feng et al., 2018). Therefore, there is a zoonotic potential for *Cryptosporidium* spp. in crab-eating macaques.

To date, 8 *G. duodenalis* assemblages (A-H) have been recognized in humans and animals, based on sequence analysis of triosephosphate isomerase (*tpi*) gene, *β*-giardin (*bg*) gene, and glutamate dehydrogenase (*gdh*) gene (Cai et al., 2021). Among 8 assemblages, assemblages A and B were most commonly found in humans and non-human primates. In contrast, assemblage E was mainly found in ruminants and occasionally found in humans (50 cases) and non-human primates (5 cases) (Brynildsrud et al., 2018; Cai et al., 2021). Previous studies have found that non-human primates were potential hosts for *G. duodenalis* (Feng and Xiao, 2011). Therefore, there has high zoonotic potential for assemblages A, B, and E of *G. duodenalis* in crab-eating macaques.

More than 500 genotypes and 15 genetic groups of *E. bieneusi* have been recognized in humans and animals, based on sequence analysis of internal transcribed spacer (ITS) gene (Li and Xiao, 2021; Li et al., 2022; Jiang et al., 2024). Among 15 groups, Group 1 was mainly found in humans and was considered zoonotic group (Li W. et al., 2019). At least 50 genotypes of *E. bieneusi* had been found in non-human primates, and these genotypes was clustered together with Group 1 (Chen et al., 2019). Genotypes A, D, Type IV, EbpC, Peru 7, Peru 8, Peru 11, BEB6, and I of *E. bieneusi* were associated with human microsporidiosis in many countries (Santín and Fayer, 2011; Wang et al., 2013; Wang et al., 2018). Among them, genotypes Type IV, Peru 8, and macaque 3 were a common genotype in humans and most animals, however, macaque3 was only detected in non-human primates in China (Karim et al., 2014b; Karim et al., 2014c; Chen et al., 2020). Therefore, there is a zoonotic potential for *E. bieneusi* in crab-eating macaques.

In addition to Beijing and Suzhou, some studies on the occurrence of *Cryptosporidium* spp., *G. duodenalis* and *E. bieneusi* in Nonhuman primates (NHPs) have also been conducted in China (Karim et al., 2014c; Karim et al., 2014d; Ye et al., 2014; Chen et al., 2019; Guo et al., 2021a; Shu et al., 2022). The crab-eating macaque farms in Beijing and Suzhou have the same history, however the scale of animals is different. The occurrence and genetic identity of *Cryptosporidium* spp., *G. duodenalis*, and *E. bieneusi* were unclear in Suzhou and Beijing. Therefore, we examined the occurrence of three pathogens in crab-eating macaques in two cities in this study. The results of the study suggest that these three intestinal pathogens were prevalent and had high zoonotic potential in these animals.

## 2 Materials and methods

### 2.1 Samples collection

A total of 504 fecal samples were collected from crab-eating macaques on commercial farms in Beijing and Suzhou, China. These farms from lab animal companies were certified by Accreditation of Laboratory Animal Care and International Association for Assessment. The crab-eating macaques farms in Beijing and Suzhou were established in 2002. The farms in Beijing and Suzhou had 8,000 and 3,000 crab-eating macaques, respectively. These two farms mainly raised young animals (< 2 months old) and adult animals (> 24 months old) in Beijing and Suzhou, and crab-eating macaques of other ages were sent off farms and some animals were dispersed to laboratories around the world. Of these fecal samples, 292 were collected from 2 convenient age groups in Suzhou, including under 2 months animals (*n* = 72) and more than 24 months animals (*n* = 227). The 212 samples from crab-eating macaques were collected from 2 convenient age groups in Beijing, including under 2 months animals (*n* = 60) and more than 24 months animals (*n* = 152). These animals investigated were divided into 2 convenient age groups: < 2 months old (*n* = 132) and > 24 months old (*n* = 379) according to the true age information of animals at the time of sampling. Crab-eating macaques <2 months represent the juvenile stage of animals, whose immune systems are not fully developed and could be more susceptible to pathogens. Crab-eating macaques >24 months reach sexual maturity, and their immune function is basically established, which could effectively deal with common pathogens. The room in which the animals are kept is cleaned every day. All crab-eating macaques had no obvious clinical signs during the sample collection period. These collected samples were stored in 2.5% potassium dichromate until DNA extraction.

### 2.2 DNA extraction

The genomic DNA of approximately 200 mg samples in crab-eating macaques were extracted using the Fast DNA Spin Kit for Soil (MP Biomedical, Santa Ana, CA, USA) as previous described (Jiang et al., 2005). The genomic DNA that had been extracted was stored at −20°C before being used in *Cryptosporidium* species, *C. hominis* subtypes, *G. duodenalis* genotypes, and *E. bieneusi* genotypes analyses.

### 2.3 Detection of *Cryptosporidium* spp., *G. duodenalis*, and *E. bieneusi*

The extracted DNA was analyzed for *Cryptosporidium* spp. by nested PCR and sequence analysis of the small subunit rRNA (*SSU* rRNA) gene (Xiao et al., 1999). The *C. hominis* identified were further subtyped by nested PCR and sequence analysis of the 60 kDa glycoprotein (*gp60*) gene (Alves et al., 2003). The *E. bieneusi* was detected by nested PCR targeting a 392-bp fragment of the internal transcribed spacer (ITS) of the rRNA gene (Sulaiman et al., 2003b). The *G. duodenalis* was detected by nested PCR targeting a 599-bp fragment of the glutamate dehydrogenase (*gdh*) gene, a 511-bp fragment of the *β*-giardin (*bg*) gene, and a 530-bp fragment of the triosephosphate isomerase (*tpi*) gene (Sulaiman et al., 2003a; Caccio and Ryan, 2008; Ye et al., 2014). Two replicates were used for PCR analysis of each sample with positive and negative samples. All primer sequences, cycling parameters, and expected products used are listed in Supplementary Table S1.

### 2.4 Sequence analysis

All positive secondary PCR products were sequenced sequenced bi-directionally in Sangon Biotech (Shanghai, China) to identify *Cryptosporidium* species*, C. hominis* subtypes, *G. duodenalis* genotypes, and *E. bieneusi* genotypes. The nucleotide sequences were assembled using ChromasPro 2.1.5.0,^1^ edited using BioEdit 7.1.3.0,^2^ and aligned using ClustalX 2.0.11.^3^ The phylogenetic relationships of the *C. hominis* subtypes and *E. bieneusi* genotypes were analysed using maximum likelihood analysis implemented in Mega 7.0^4^ based on substitution rates calculated with the general time reversible model as described (Yan et al., 2017).

### 2.5 Statistical analysis

Detection rates of *Cryptosporidium* species, *G. duodenalis*, and *E. bieneusi* were compared between different age groups and cities using the Chi-square test implemented in SPSS v.20.0 (IBM Corp., New York, NY, USA). Differences were considered significant at *p* < 0.05.

## 3 Results

### 3.1 Occurrence of *Cryptosporidium* spp. in crab-eating macaques

Of the 504 fecal samples collected from crab-eating macaques, the detection rate of *Cryptosporidium* spp. was 11.9% (60/504) in Suzhou and Beijing in present study. The 15.1% (44/292) detection rate of *Cryptosporidium* spp. from crab-eating macaques in Suzhou was significantly higher than that in Beijing (2.8%; 6/212; *χ*^2^ = 20.6, *df* = 1, *p* < 0.0001; Table 1). By age, the *Cryptosporidium* detection rates in crab-eating macaque of < 2 months and > 24 months were 32.6% (43/132) and 1.8% (7/379), respectively. The detection rate of *Cryptosporidium* spp. was significant different in two age groups (*χ*^2^ = 104.7, *df* = 1, *p* < 0.0001; Table 2).

**Table 1:** Distribution of *Cryptosporidium* spp., *G. duodenalis*, and *E. bieneusi* in crab-eating macaques, China.

Location	Age (months)	No. specimens	*Cryptosporidium* spp.			*G. duodenalis*		*E. bieneusi*	
			No. positive (%)	*C. hominis* (*n*)	Subtype (*n*)	No. positive (%)	Genotype (*n*)	No. positive (%)	Genotype (*n*)
Suzhou	< 2	72	39 (54.2)	39	IbA13G4 (24); InA26 (3); IeA11G3T3 (2); IfA17G2R3 (2)	13 (18.1)	B (13)	8 (11.1)	CM1 (4); Peru8 (3); D (1)
	> 24	227	5 (2.2)	5	IbA13G4 (3); IfA17G2R3 (1)	15 (6.6)	B (15)	14 (6.2)	CM1 (10); Peru8 (2); D (2)
	Subtotal	292	44 (15.1)	44	IbA13G4 (27); InA26 (3); IeA11G3T3 (2); IfA17G2R3 (3)	28 (8.6)	B (28)	22 (7.5)	CM1 (14); Peru8 (5); D (3)
Beijing	< 2	60	4 (6.7)	4	IiA17 (2)	0 (0.0)	-	1 (1.7)	Type IV (1)
	> 24	152	2 (1.3)	2	IiA17 (1)	0 (0.0)	-	0 (0.0)	-
	Subtotal	212	6 (2.8)	6	IiA17 (3)	0 (0.0)	-	1 (0.5)	Type IV (1)
Total		504	60 (11.9)	60	IbA13G4 (27); InA26 (3); IeA11G3T3 (2); IfA17G2R3 (3); IiA17 (3)	28 (5.6)	B (28)	23 (4.6)	CM1 (14); Peru8 (5); D (3); Type IV (1)

**Table 2:** Occurrence of *Cryptosporidium* spp., *G. duodenalis*, and *E. bieneusi* in crab-eating macaques in China by age.

Age (months)	No. specimens	*Cryptosporidium* spp.			*G. duodenalis*		*E. bieneusi*	
		No. positive (%)	*C. hominis* (*n*)	Subtype (*n*)	No. positive (%)	Genotype (*n*)	No. positive (%)	Genotype (*n*)
< 2	132	43 (32.6)	43	IbA13G4 (24); InA26 (3); IeA11G3T3 (2); IfA17G2R3 (2); IiA17 (2)	13 (9.8)	B (13)	9 (6.8)	CM1 (4); Peru8 (3); D (1); Type IV (1)
> 24	379	7 (1.8)	7	IbA13G4 (3); IfA17G2R3 (1); IiA17 (1)	15 (4.0)	B (15)	14 (3.7)	CM1 (10); Peru8 (2); D (2)
Total	504	60 (11.9)	60	IbA13G4 (27); InA26 (3); IeA11G3T3 (2); IfA17G2R3 (3); IiA17 (3)	28 (5.6)	B (28)	23 (4.6)	CM1 (14); Peru8 (5); D (3); Type IV (1)

**Figure 1:**
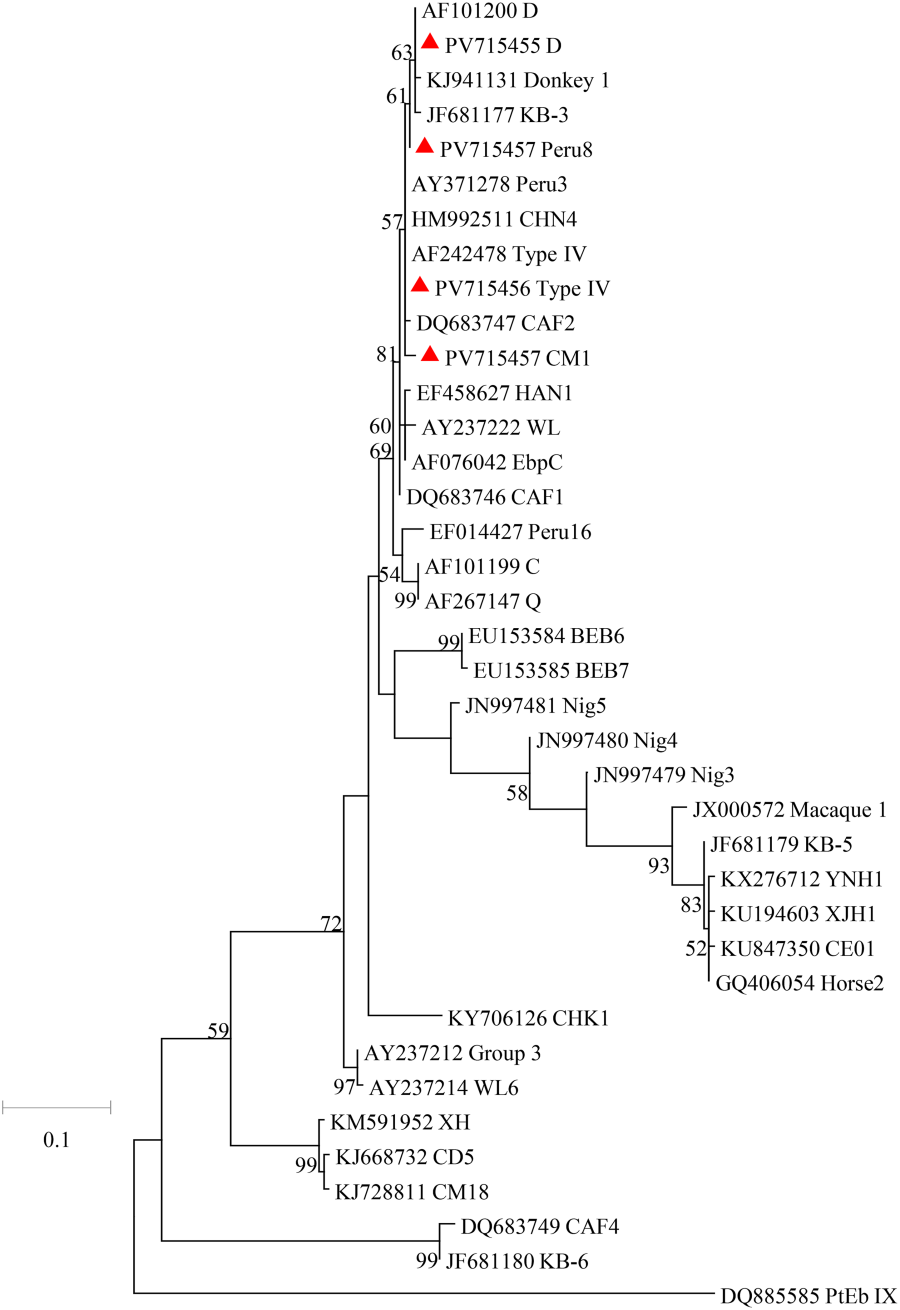
Phylogenetic relationships of *C. hominis* subtypes based on maximum likelihood analysis. The subtypes of *C. hominis* that have been identified in this study are indicated by red triangles. Bootstrap values below 50% are not shown. Bar = 0.5 substitutions per site.

A total of 60 *Cryptosporidium*-positive samples were successfully sequenced based on the *SSU* rRNA gene. Only one *Cryptosporidium* species was identified, namely *C. hominis* (*n* = 60). The *SSU* rRNA gene sequences of *C. hominis* generated in this study had a single nucleotide variation from the reference sequences reported from *Macaca mulatta* (GenBank: ON023862). Of the 60 *C. hominis* samples, five subtypes of five subtype families were successfully identified at the *gp60* gene: IbA13G4 (*n* = 27), InA26 (*n* = 3), IfA17G2R3 (*n* = 3), IiA17 (*n* = 3), and IeA11G3T3 (*n* = 2). The sequences from subtypes IfA17G2R3, IeA11G3T3, and IiA17 were identical to the reference sequence ON036042 from *Macaca mulatta*, AY738184 from children, and MK952706 from *Macaca fascicularis*, respectively. The sequences from subtypes IbA13G4 had TCA and TCG difference in compared to the reference sequence MK982515 from rhesus macaque. In contrast, the *gp*60 sequence of the InA26 had 9 single nucleotide polymorphisms (SNPs) compared to the reference sequence MG952711 obtained from *Macaca fascicularis*. In phylogenetic analysis of the *C. hominis* subtypes obtained from the study, emerging subtype IbA13G4 clustered with other If subtypes (Figure 1).

### 3.2 Occurrence of *G. duodenalis* in crab-eating macaques

In present study, the detection rate of *G. duodenalis* was 5.6% (28/504) in Suzhou and Beijing. The 8.6% (28/292) detection rate of *G. duodenalis* from crab-eating macaques in Suzhou was significantly higher than that in Beijing (0.0%; 0/212; *χ*^2^ = 21.5, *df* = 1, *p* < 0.0001; Table 1). By age, the *G. duodenalis* detection rates in crab-eating macaque of < 2 months and > 24 months were 9.8% (13/132) and 4.0% (15/379), respectively. The detection rate of *G. duodenalis* were significant different in two age groups (*χ*^2^ = 6.6, *df* = 1, *p* = 0.0104; Table 2).

The secondary PCR products from 28 *G. duodenalis* positive samples had been successfully sequenced. Only assemblage B was identified in these positive samples in Suzhou. The obtained sequences from assemblage B samples were identical to the GenBank reference sequence MK262843 from crab-eating macaque.

### 3.3 Occurrence of *E. bieneusi* in crab-eating macaques

In present study, the detection rate of *E. bieneusi* was 4.6% (23/504) in Suzhou and Beijing. The 7.5% (22/292) detection rate of *E. bieneusi* from crab-eating macaques in Suzhou was significantly higher than that in Beijing (0.5%; 1/212; *χ*^2^ = 14.0, *df* = 1, *p* = 0.0001; Table 1). By age, the *E. bieneusi* detection rates in crab-eating macaque of < 2 months and > 24 months were 6.8% (9/132) and 3.7% (14/379), respectively. There were no significant different in the detection rate of *E. bieneusi* in two age groups (*χ*^2^ = 2.2, *df* = 1, *p* = 0.1360; Table 2).

**Figure 2:**
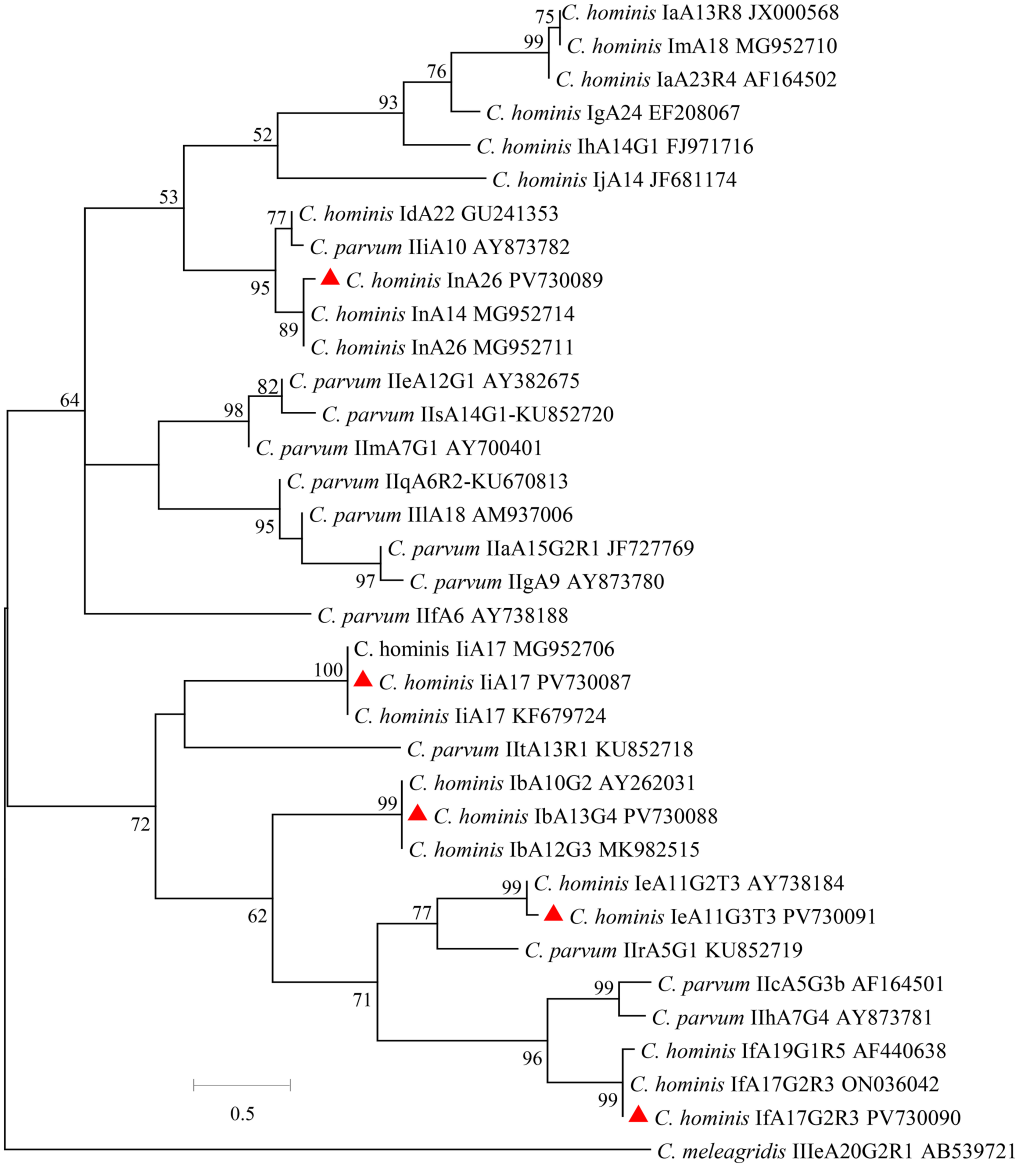
Phylogenetic relationships of *E. bieneusi* genotypes based on maximum likelihood analysis. The genotypes of *E. bieneusi* that have been identified in this study are indicated by red triangles. Bootstrap values below 50% are not shown. Bar = 0.1 substitutions per site.

The ITS products from 23 *E. bieneusi*-positive specimens from crab-eating macaques were sequenced successfully. A total of 4 known genotypes were detected, including CM1 (*n* = 14), Peru8 (*n* = 5), D (*n* = 3), and Type IV (*n* = 1). Among them, CM1 was the dominant genotype in Suzhou, while only one genotype was found in Beijing. The sequences from genotypes CM1, Peru8, D, and Type IV were identical to the reference sequence KF305581 from Rhesus macaque, JF927959 from chicken, MT895457 from amur tiger, and AF242478 from human, respectively. In the maximum likelihood analysis of the *E. bieneusi* genotypes, genotypes CM1, Peru8, D, and Type IV were clustered with Group 1 (Figure 2).

## 4 Discussion

The results of this study indicate that *Cryptosporidium* spp., *G. duodenalis*, and *E. bieneusi* are prevalent in crab-eating macaques in Suzhou and Beijing of Chinese cities. Altogether, the detection rates for *Cryptosporidium* spp., *G. duodenalis*, and *E. bieneusi* were 11.9, 5.6, and 4.6%, respectively. The detection rate (11.9%) in this study for *Cryptosporidium* spp. was higher than that observed in free-range monkeys conducted in Shanxi (3.0%), Yunan (0 and 0.6%), Guangxi (1.0%), and Guizhou (0.7%) of China (Karim et al., 2014d; Du et al., 2015; Gu et al., 2016; Jia et al., 2022; Shu et al., 2022). However, it is similar to the prevalence in farmed crab-eating macaques in Hainan (9.1%) (Chen et al., 2019). The high detection rate of *Cryptosporidium* spp. in this study may be due to the highly intensive farming model in crab-eating macaque farms. Further comparison with other countries, the detection rate of farmed crab-eating macaques was also higher than that free-range NHPs in Thailand (1.0%), Kenya (2.6%), Madagascar (4.0%), and Rwanda (4.0%) (Li et al., 2011; Sak et al., 2014; Bodager et al., 2015; Sricharern et al., 2016). Therefore, the intensive farming of animals was conducive to the transmission of *Cryptosporidium* spp. The detection rates of *G. duodenalis* (5.6%) and *E. bieneusi* (4.6%) in this study are lower than those found in NHPs in other studies, which reported detection rates ranging from 8.5 to 32.3% for *G. duodenalis* and from 11.4 to 46.2% for *E. bieneusi* (Johnston et al., 2010; Beck et al., 2011; Ye et al., 2012; Du et al., 2015; Karim et al., 2015; Zhong et al., 2017). The low detection rate of these two pathogens may be due to the high prevalence of *Cryptosporidium* limit the transmission of them. Among the two cities, the detection rates of *Cryptosporidium* spp., *G. duodenalis*, and *E. bieneusi* were higher in Suzhou, probably because of the higher stocking density on this farm. By age, the detection rates for *Cryptosporidium* spp. (32.6%), *G. duodenalis* (9.8%), and *E. bieneusi* (6.8%) in monkeys of under 2 months of age were higher than those over 2 years (1.8, 4.0%; 3.7%). Similar results have been found in other animals, including bamboo rats and horses (Li F. et al., 2019; Li et al., 2020). This may be related to the relatively low immunity of young crab-eating macaques.

The *C. hominis* subtypes found in this study belongs to highly divergent subtypes. Five subtypes of *C. hominis* were identified in crab-eating macaques in this study, namely IbA13G4 (*n* = 27), InA26 (*n* = 3), IiA17 (*n* = 3), IfA17G2R3 (*n* = 3), and IeA11G3T3 (*n* = 2). The emerging subtype IbA13G4 was dominant subtypes in this study, and was detected in crab-eating macaques for the first time. In previous studies, many outbreaks of cryptosporidiosis were caused by Ib subtype family around the world (Yang et al., 2021; Huang et al., 2025). The subtype IbA10G2 is responsible for most outbreaks of cryptosporidiosis in humans in both industrialized and developing countries (Cacciò and Chalmers, 2016; Feng et al., 2018). Furthermore, previous studies have shown that IbA12G3 induced a significantly higher intensity of oocyst and had higher parasite loads in the mouse intestine (Huang et al., 2024). Similarly, the Ie, If, and Ii subtype families are common in humans worldwide (Xiao and Feng, 2017). Among them, subtypes IeA11G3T3 and IiA17 were occasionally found in cancer patients and HIV-infected patients, they are apparently zoonotic (Sannella et al., 2019; Makipour et al., 2025). In contrast, the subtype IfA17G2R3 and InA26 were only found in rhesus monkeys in Guizhou and in crab-eating macaques in Hainan, respectively (Chen et al., 2019; Jia et al., 2022). Therefore, these two subtypes have potential zoonotic risk. In the future, we will conduct more studies to evaluate the infectivity and pathogenicity of *C. hominis* subtypes in animals.

The crab-eating macaques could be reservoirs for zoonotic assemblage B. Similar to other studies in crab-eating macaques, only assemblage B was found in this study (Karim et al., 2014d; Cai et al., 2021). Previous studies have shown that assemblage B had the broadest host range, and assemblage B was responsible for most giardiasis cases in humans (Feng and Xiao, 2011). In contrast, the assemblage A and E were occasionally found in non-human primates. A few studies had shown that the assemblage A was found in some non-human primates in China (Karim et al., 2014d; Ye et al., 2014) and other countries (Sricharern et al., 2016; Brynildsrud et al., 2018). In addition, assemblage E were found in five non-human primates (Brynildsrud et al., 2018). In present study, assemblage B was the only assemblage in the crab-eating macaques. This could have been due to the confined nature of animals in the facility, which limits the introduction of other genotypes. The common occurrence of assemblage B suggested that *G. duodenalis* from crab-eating macaques has high zoonotic potential.

Crab-eating macaques may potentially contribute to the zoonotic transmission of *E. bieneusi* genotypes to humans. In this study, 4 *E. bieneusi* genotypes were found, namely CM1 (14 specimens), Peru8 (5 specimens), D (3 specimens), and Type IV (1 specimen) and these genotypes belong to the zoonotic Group 1. Among these genotypes of *E. bieneusi*, genotypes D, Peru 8, and Type IV are mainly identified in humans, and have been frequently documented in domestic and wild animals, including non-human primates (Li W. et al., 2019; Li and Xiao, 2021; Li et al., 2022). In previous studies, the genotype CM1 has been only found in non-human primates in Guangdong, Guangxi, Yunan, and Sichuan of China and it was not found in humans (Karim et al., 2014a; Karim et al., 2014b). This is probably because only a small number of studies have been performed on human *E. bieneusi* infection in China. The common occurrence of zoonotic genotypes suggested that *E. bieneusi* from crab-eating macaques has high zoonotic potential.

## 5 Conclusion

This study reported the prevalence of *Cryptosporidium* spp., *G. duodenalis*, and *E. bieneusi* in crab-eating macaques in Beijing and Suzhou cities, China. The results indicate that known zoonotic *C. hominis*, Assemblage B, and *E. bieneusi* genotypes are prevalent in crab-eating macaques. Crab-eating macaques are in close contact with humans. Therefore, crab-eating macaques may play a potential role in the zoonotic transmission of pathogens to humans. Further studies are needed to monitor the molecular epidemiology of these three pathogens in farmed crab-eating macaques.

## Data Availability

The datasets presented in this study can be found in online repositories. The names of the repository/repositories and accession number(s) can be found here: https://www.ncbi.nlm.nih.gov/genbank/, PV730087-PV730091 and PV715454-PV715457.

## Footnotes

^1^ http://technelysium.com.au/ChromasPro.html

^2^ https://bioedit.software.informer.com

^3^ http://clustal.org

^4^ http://www.megasoftware.net
