# Supplementary material for: Distribution of human-pathogenic Cryptosporidium spp., Giardia duodenalis, and Enterocytozoon bieneusi in crab-eating macaques in China
Source: Front Microbiol. 2025 Jul 21;16:1641632. doi: 10.3389/fmicb.2025.1641632 (PMC12318935; doi:10.3389/fmicb.2025.1641632)
Supplement: Supplementary file 1 [file Table_1.docx]

**Distribution of human-pathogenic *Cryptosporidium* spp., *Giardia duodenalis*, and *Enterocytozoon bieneusi* in crab-eating macaques in China**

Huilin Zhang^1^, Huiyang Chen^1^, Chaoyue He^1^, Wenchao Li^2^,* and Falei Li^1^,*

^1^ Anhui Province Key Laboratory of Embryo Development and Reproductive Regulation, College of Biological and Food Engineering, Fuyang Normal University, Fuyang, 236037, China

^2^ Anhui Province Key Laboratory of Animal Nutritional Regulation and Health, College of Animal Science, Anhui Science and Technology University, Fengyang, 233100, China.

*Correspondence to: Wenchao Li ([liwen303@126.com](mailto:liwen303@126.com)); Falei Li (fli@fynu.edu.cn)

**Supplementary Table S1:** Primer sequences, and PCR conditions used in this study.

| **Target gene** | **Primer sequences** | **PCR conditions** | **Products (bp)** |
| --- | --- | --- | --- |
| *SSU* rRNA | F1: TTCTAGAGCTAATACATGCG | 94°C - 5 min (35x)  55°C - 45 s  68°C - 1 min | 830 |
|  | R1: CCCATTTCCTTCGAAACAGGA |  |  |
|  | F2: GGAAGGGTTGTATTTATTAGATAAAG | 94°C - 5 min (35x)  55°C - 45 s  68°C - 1 min |  |
|  | R2: CTCATAAGGTGCTGAAGGAGTA |  |  |
| *gp60* | F1: TTACTCTCCGTTATAGTCTCC | 94°C - 5 min (35x)  52°C - 45 s  68°C - 1 min | 800 |
|  | R1: GGAAGGAACGATGTATCTGA |  |  |
|  | F2: TCCGCTGTATTCTCAGCC | 94°C - 5 min (35x)  50°C - 45 s  68°C - 1 min |  |
|  | R2: GCAGAGGAACCAGCATC |  |  |
| *gdh* | F1: TTCCGTGTCCAGTACAACTC | 94°C - 5 min (35x)  50°C - 45 s  68°C - 1 min | 392 |
|  | R1: GCCAGCTTCTCCTCGTTGAA |  |  |
|  | F2: CGCTTCCACCCCTCTGTCAAT | 94°C - 5 min (35x)  50°C - 45 s  68°C - 1 min |  |
|  | R2: TGTTGTCCTTGCACATCTC |  |  |
| *bg* | F1: AAGCCCGACGACCTCACCCGCAGTGC | 94°C - 5 min (35x)  65°C - 45 s  68°C - 1 min | 510 |
|  | R1: GAGGCCGCCCTGGATCTTCGAGACGAC |  |  |
|  | F2: GAACGAGATCGAGGTCCG | 94°C - 5 min (35x)  55°C - 45 s  68°C - 1 min |  |
|  | R2: CTCGACGAGCTTCGTGTT |  |  |
| *tpi* | F1: AATAAATIATGCCTGCTCGTCG | 94°C - 5 min (35x)  54°C - 45 s  68°C - 1 min | 530 |
|  | R1: ATGGACITCCTCTGCCTGCTC |  |  |
|  | F2: CCCTTCATCGGIGGTAACTTCAA | 94°C - 5 min (35x)  58°C - 45 s  68°C - 1 min |  |
|  | R2: GTGGCCACCACICCCGTGCC |  |  |
| ITS | F1: GATGGTCATAGGGATGAAGAGCTT | 94°C - 5 min (35x)  55°C - 45 s  68°C - 1 min | 400 |
|  | R1: TATGCTTAAGTCCAGGGAG |  |  |
|  | F2: AGGGATGAAGAGCTTCGGCTCTG | 94°C - 5 min (35x)  55°C - 45 s  68°C - 1 min |  |
|  | R2: AGTGATCCTGTATTAGGGATATT |  |  |
